# Identification of Embryonic Chicken Proteases Activating Newcastle Disease Virus and Their Roles in the Pathogenicity of Virus Used as *In Ovo* Vaccine

**DOI:** 10.1128/jvi.00324-23

**Published:** 2023-04-12

**Authors:** Helong Feng, Lun Yao, Zhe Zeng, Liren Jiang, Yu Shang, Hongcai Wang, Li Li, Zichen Wang, Xin Wang, Hongchun Yang, Qingqing Zhao, Xiangfei Ren, Tengfei Zhang, Rongrong Zhang, Yunqing Guo, Qin Lu, Qiao Hu, Wenting Zhang, Chan Ding, Huabin Shao, Guofu Cheng, Qingping Luo, Guoyuan Wen

**Affiliations:** a Institute of Animal Husbandry and Veterinary Sciences, Hubei Academy of Agricultural Sciences, Wuhan, China; b Key Laboratory of Prevention and Control Agents for Animal Bacteriosis, Ministry of Agriculture, Wuhan, China; c Hubei Provincial Key Laboratory of Animal Pathogenic Microbiology, Wuhan, China; d Division of Veterinary Pathology, College of Veterinary Medicine, Huazhong Agricultural University, Wuhan, China; e Department of Avian Diseases, Shanghai Veterinary Research Institute, Chinese Academy of Agricultural Sciences, Shanghai, China; f Hubei Hongshan Laboratory, Wuhan, China; University of Kentucky College of Medicine

**Keywords:** Newcastle disease virus, *in ovo* vaccination, pathogenicity, tissue tropism, fusion protein cleavage site, trypsin-like protease

## Abstract

*In ovo* vaccination is an attractive immunization approach for chickens. However, most live Newcastle disease virus (NDV) vaccine strains used safely after hatching are unsafe as *in ovo* vaccines due to their high pathogenicity for chicken embryos. The mechanism for viral pathogenicity in chicken embryos is poorly understood. Our previous studies reported that NDV strain TS09-C was a safe *in ovo* vaccine, and the F protein cleavage site (FCS) containing three basic amino acids (3B-FCS) was the crucial determinant of the attenuation of TS09-C in chicken embryos. Here, five trypsin-like proteases that activated NDV in chicken embryos were identified. The F protein with 3B-FCS was sensitive to the proteases Tmprss4, Tmprss9, and F7, was present in fewer tissue cells of chicken embryos, which limited the viral tropism, and was responsible for the attenuation of NDV with 3B-FCS, while the F protein with FCS containing two basic amino acids could be cleaved not only by Tmprss4, Tmprss9, and F7 but also by Prss23 and Cfd, was present in most tissue cells, and thereby was responsible for broad tissue tropism and high pathogenicity of virus in chicken embryos. Furthermore, when mixed with the protease inhibitors aprotinin and camostat, NDV with 2B-FCS exhibited greatly weakened pathogenicity in chicken embryos. Thus, our results extend the understanding of the molecular mechanism of NDV pathogenicity in chicken embryos and provide a novel molecular target for the rational design of *in ovo* vaccines, ensuring uniform and effective vaccine delivery and earlier induction of immune protection by the time of hatching.

**IMPORTANCE** As an attractive immunization approach for chickens, *in ovo* vaccination can induce a considerable degree of protection by the time of hatching, provide support in closing the window in which birds are susceptible to infection, facilitate fast and uniform vaccine delivery, and reduce labor costs by the use of mechanized injectors. The commercial live Newcastle disease virus (NDV) vaccine strains are not safe for *in ovo* vaccination and cause the death of chicken embryos. The mechanism for viral pathogenicity in chicken embryos is poorly understood. In the present study, we identified five trypsin-like proteases that activate NDV in chicken embryos and elucidated their roles in the tissue tropism and pathogenicity of NDV used as *in ovo* vaccine. Finally, we revealed the molecular basis for the pathogenicity of NDV in chicken embryos and provided a novel strategy for the rational design of *in ovo* ND vaccines.

## INTRODUCTION

Newcastle disease (ND) is one of the most important avian diseases due to its likelihood of devastating economic losses (1). Newcastle disease virus (NDV), the causative agent of ND, has been classified into lentogenic, mesogenic, and velogenic pathotypes based on the viral pathogenicity in chickens (2). Birds infected with velogenic NDV breed severe disease, characterized by typical respiratory and neurological signs with high mortality (3). NDV as an enveloped virus with a nonsegmented, single-stranded RNA genome of negative polarity belongs to the genus *Avaluvirus* within the family *Paramyxoviridae*. The 15.2-kb genome encodes six structural proteins, the nucleoprotein (NP), phosphoprotein (P), matrix (M), fusion (F), hemagglutinin-neuraminidase (HN), and large polymerase protein (L), as well as two other nonstructural proteins, V and W (4). Viral particles contain two surface glycoproteins, HN and F, embedded in a lipid membrane. The HN protein is a multifunctional protein involved in the recognition of sialic acid receptors on the cell surface, the removal from receptors, and the interaction with F protein to promote the fusion of the virion envelope with the host cell plasma membrane (5, 6).

Immunization with live vaccines via conventional approaches, such as eye-drop, drinking water, and aerosol, serves as a common strategy for ND control in many countries (7). As an attractive immunization approach for chickens, *in ovo* vaccination can induce a considerable degree of protection by the time of hatching, provide support in closing the window in which birds are susceptible to infection, facilitate fast and uniform vaccine delivery, and reduce labor costs by the use of mechanized injectors (8, 9). At present, vaccines for several avian diseases, such as Marek’s disease, infectious bursal disease (IBD), and fowl pox, have been approved for *in ovo* immunization (9). Currently, *in ovo* immunization against Marek’s disease has been used in over 90% of U.S. hatcheries. For NDV, although commercial live vaccine strains such as LaSota, V4, and Clone 30 have been used safely and efficiently in chickens after hatching, most of them are not safe for *in ovo* vaccination and result in poor survival and severe histopathological lesions in hatched birds (10–12).

New methods that have been proposed may be used to weaken the pathogenicity of ND vaccine strains in chicken embryos. First, strain Hitchner B1 mixed with NDV neutralizing antibody showed greatly weakened pathogenicity in chicken embryos (13). However, it was difficult to standardize such vaccines. Second, by replacing the L gene with that of strain Clone 30 and by inserting the VP2 gene of IBD virus (IBDV), the chimeric strain LaSota was attenuated significantly in chicken embryos and therefore was a safe and effective *in ovo* vaccine against both NDV and IBDV (14). Third, with serial passaging in heterologous BHK-21 cells, the TS09-C strain derived from strain V4 (15) was safe and immunogenic as an *in ovo* vaccine for both specific-pathogen-free (SPF) and commercial chicken embryos (7, 16). It had been demonstrated that the attenuation of strain TS09-C in chicken embryos was determined by the F protein cleavage site (FCS) containing three basic amino acids (17).

The pathogenicity of NDV in chickens after hatching has been well documented. The F protein plays a major role in the virulence of NDV for hatched birds. F protein is synthesized as a fusion-incompetent precursor protein, F0, in the infected cells and requires cleavage by host cellular proteases into subunits F1 and F2. Cleavage of F protein is a prerequisite for triggering membrane fusion and is essential for virus infectivity (1, 18). Low-virulence NDV strains contain two basic amino acids in its FCS (2B-FCS), rendering the F protein insensitive to the intracellular proteases, and depend on the extracellular trypsin-like proteases to be cleaved, limiting viral tropism to the respiratory and digestive tracts, while the high-virulence strains with four basic amino acids in their FCS act as preferred recognition sites for furin-like proteases present in most tissue cells (19). The cleavage of F protein in a wide range of tissue cells is responsible for the systemic replication and broad tissue tropism of NDV as well as its high virulence.

The pathogenicity of NDV in chicken embryos has been studied extensively, but the mechanism for viral pathogenicity in birds before hatching is poorly understood. In this study, by using strains TS09-C and rTS-2B as the model, the mechanism for pathogenicity of NDV in chicken embryos was elucidated. The F protein with FCS containing three basic amino acids (3B-FCS) was sensitive to the cellular trypsin-like proteases Tmprss4, Tmprss9, and F7, present in fewer tissue cells of chicken embryos, which limited the viral tissue tropism, and was responsible for the attenuation of NDV, while the F protein with 2B-FCS could be cleaved not only by Tmprss4, Tmprss9, and F7 but also by Prss23 and Cfd, present in most tissue cells and responsible for the broad tissue tropism and high virulence of NDV in chicken embryos.

## RESULTS

### Pathogenicity of NDV in chicken embryos is correlated with viral tissue tropism.

**FIG 1:**
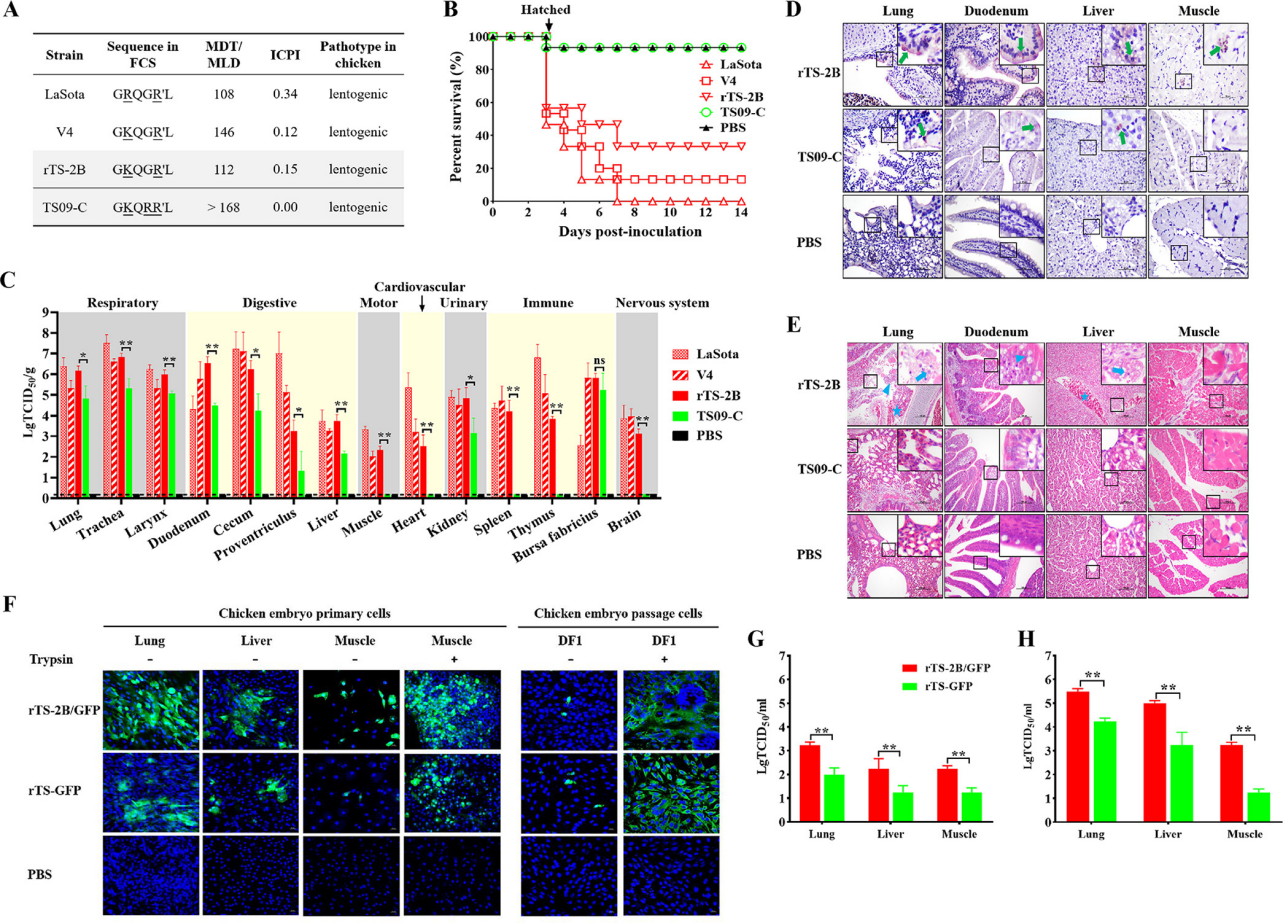
Pathogenicity of NDV in chicken embryos and its relationship with viral tissue tropism. (A) Pathogenic characterization of NDV strains LaSota, V4, rTS-2B, and TS09-C in chickens. (B) Percentages of survival of chicken embryos vaccinated *in ovo* with NDV strains. (C) Viral titers in the 14 kinds of tissues collected from chickens inoculated *in ovo* with different NDV strains at 3 dpi. The viral titer of each sample was determined in BHK-21 cells. Statistical significance of the viral titer was determined with two-tailed *t* tests (nonsignificant [ns], *P* > 0.05; *, 0.01 < *P* < 0.05; **, *P* < 0.01). (D) IHC detection of NDV in tissue samples from *in ovo*-vaccinated birds at 3 dpi. The tissue samples were fixed in 4% paraformaldehyde, paraffin embedded, sectioned, stained with chicken anti-NDV antibody, and analyzed microscopically. The green arrows indicate NDV-positive signals. (E) Histopathological analysis of tissue samples from chickens vaccinated *in ovo* with different NDV strains at 3 dpi. The lesions are indicated by different symbols (arrows, cell necrosis; triangles, lymphocyte infiltration or goblet cell proliferation; stars, hemorrhage or congestion). (F) Fluorescence microscopy analysis of GFP expression in chicken embryonic primary and passage cells infected by rTS-2B/GFP and rTS-GFP (MOI, 0.001), with or without the addition of TPCK-trypsin (0.2 μg/mL) at 48 hpi. (G and H) Viral titers in chicken embryonic primary cells infected by rTS-2B/GFP and rTS-GFP (MOI, 0.1) without TPCK-trypsin at 24 hpi (G) and 48 hpi (H). Lg, log10. Media from infected cells were harvested at 24 hpi (G) and 48 hpi (H), and viral titers were determined by TCID_50_ titration in BHK-21 cells.

To explore the mechanism for pathogenicity of NDV in chicken embryos, we investigated the pathogenicity and tissue tropism of four lentogenic NDV strains with different FCSs in chicken embryos. Three of them possessed 2B-FCS, including strains LaSota, V4, and rTS-2B (R115G mutation based on the backbone of TS09-C strain) (17), while the TS09-C strain possessed 3B-FCS (Fig. 1A). Three NDVs with 2B-FCS displayed high pathogenicity, and the survival rates of birds at 14 days postinoculation (dpi) were lower than 37.5%, while strain TS09-C with 3B-FCS exhibited low pathogenicity with a survival rate of 93.3% (Fig. 1B). Similar results were obtained from the body weight gain test (see Fig. S1 in the supplemental material). Three strains with 2B-FCS could replicate in all 14 tissues covering seven main tissue systems, while strain TS09-C replicated only in some of the tissues (9/14). The titers of strain TS09-C in most tissues (13/14) were significantly lower than those of strain rTS-2B (Fig. 1C; Fig. S2). Fewer NDV-positive signals were observed in the lung, trachea, and duodenum tissues in the TS09-C group than in the rTS-2B group. The NDV-positive signals were not observed in the muscle tissue in the TS09-C group (Fig. 1D). Histopathological lesions were not observed in the TS09-C and phosphate-buffered saline (PBS) group (see Materials and Methods). But in the rTS-2B group, the birds displayed moderate histopathological lesions in the lung (hemorrhage, necrocytosis, and lymphocyte infiltration), duodenum (shortening of intestinal villi and goblet cell proliferation), and liver (congestion and hepatocyte necrosis) (Fig. 1E). These results revealed that the pathogenicity of NDV in chicken embryos was correlated with viral tissue tropism.

To further confirm the replication of NDV in these tissues, three kinds of primary tissue cells (lung, liver, and muscle) were cultured *in vitro*, and the replication of NDV strains rTS-2B/GFP (GFP genes inserted into the P-M gene of TS-2B) and rTS-GFP (see Materials and Methods) in these cells was evaluated. In the absence of trypsin, rTS-2B/GFP was able to replicate in all three kinds of primary cells, while rTS-GFP was able to replicate in lung and liver cells but not in muscle cells. However, in the presence of trypsin, rTS-GFP replicated efficiently in the primary muscle cells (Fig. 1F), which indicated that the proteases activating rTS-GFP were expressed in the lung and liver tissues but not in the muscle. The replication titers of rTS-2B/GFP in primary cells were significantly higher than those of rTS-GFP (Fig. 1G and H). These results were consistent with those from the viral tissue tropism test.

### Differences in chicken embryo tissues in the expression and distribution of trypsin-like serine proteases.

**FIG 2:**
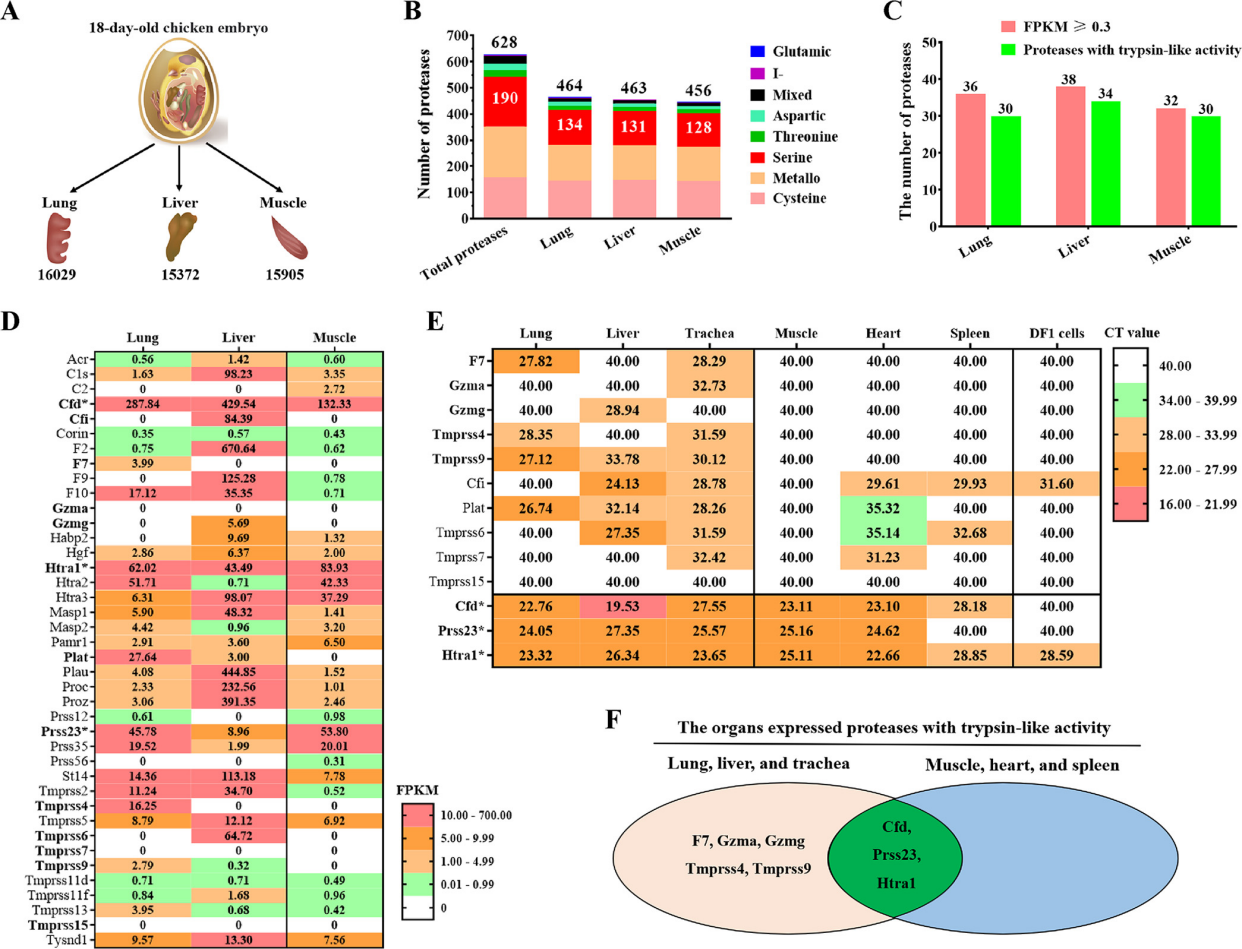
Protease transcriptome analysis of the lung, liver, and muscle tissues from 18-day-old chicken embryos by RNA-seq. (A) The total numbers of all detected genes in lung, liver, and muscle tissues from chicken embryos are indicated below the diagrams of tissues. (B) Numbers and classification of all detected proteases in the three tissues from chicken embryos. The numbers of all detected proteases and serine proteases in corresponding tissues are indicated above the bar and in the bar (red region), respectively. (C) The numbers of all detected trypsin-like serine proteases in chicken embryonic tissues, with substrate preference for an arginine residue in P1 based on the UniProt and MEROPS databases. (D) Heat map of detected trypsin-like proteases expressed in lung, liver, and muscle tissues from chicken embryos, with at least one FPKM value of ≥0.3 and a tag count of ≥50 out of three biological replicates. The mean FPKM values with color-coded expression levels (red, high expression; green, low expression) are shown. (E) Heat map showed the expression levels of selected trypsin-like proteases in six tissues (lung, liver, trachea, muscle, heart, and spleen) from chicken embryos and DF1 cells. The expression levels of proteases were determined by RT-qPCR assay, and the mean *C_T_* values with color-coded expression levels (red, high expression; green, low expression) are shown as indicated in the key. (F) Venn diagram showing the differently expressed proteases with trypsin-like activities in two groups of tissues from chicken embryos. One group included lung, liver, and trachea, and the other group included muscle, heart, and spleen.

To further screen for the NDV-activating protease candidates, we analyzed the protease repertoire of lung, liver, and muscle tissues from chicken embryo by using RNA sequencing (RNA-seq). First, the total numbers of detected genes were 16,029 in lung, 15,372 in liver, and 15,905 in muscle (Fig. 2A). Second, the numbers of genes for proteases and homologs expressed in lung, liver, and muscle were 464, 463, and 456, respectively. Of these, 134 (lung), 131 (liver), and 128 (muscle) expressing serine protease genes were detected (Fig. 2B). Third, the trypsin-like serine proteases had a substrate preference for an arginine residue in the P1 position, which was consistent with the FCS of NDV. Thus, the detected serine proteases were analyzed with the UniProt (20) and MEROPS (21) databases, and 30 expressed trypsin-like proteases were selected in lung and muscle and 34 were detected in liver (Fig. 2C). Fourth, the expression profiles of these trypsin-like proteases greatly differed among these three tissues (Fig. 2D). The NDV with 3B-FCS could replicate in the lung and liver tissues but not in muscle tissue. Therefore, we focused on the trypsin-like proteases that were not expressed in muscle but were expressed in lung or liver tissues, and then a total of 10 trypsin-like proteases were selected, including Cfi, F7, Gzma, Gzmg, Plat, Tmprss4, Tmprss6, Tmprss7, Tmprss9, and Tmprss15. The expression of 10 proteases in these three tissues and another three tissues (trachea, heart, and spleen) was further confirmed by using a reverse transcription-quantitative PCR (RT-qPCR) assay. Because the NDV with 3B-FCS could not replicate in the heart and spleen tissues, four proteases (Cfi, Plat, Tmprss6, and Tmprss7) expressed in the two tissues were excluded. In addition, the expression of Tmprss15 was not detected in all of the tissues and was therefore excluded (Fig. 2E). Finally, the remaining five proteases (F7, Gzma, Gzmg, Tmprss4, and Tmprss9) were confirmed to be expressed in the lung, liver, and trachea tissues but not in the muscle, heart, and spleen tissues (Fig. 2F) and were considered the candidate proteases activating the NDV with 3B-FCS. Moreover, three proteases (Cfd, Htra1, and Prss23) were found to be highly expressed in most of these tissues (Fig. 2D and E) and were considered the candidate proteases activating the NDV with 2B-FCS (Fig. 2F).

### Identification of trypsin-like serine proteases that activate NDV in DF1 cells.

In DF1 cells, the replication of rTS-GFP and rTS-2B/GFP was confirmed to be dependent on the addition of trypsin (Fig. 1F). DF1 cells also did not express most of the candidate proteases activating NDV with 3B-FCS (5/5) and 2B-FCS (2/3), with the exception of Htra1 (Fig. 2E), indicating that it was not an NDV-activating protease. Thus, DF1 cells were selected as a cell model to evaluate the effects of candidate proteases on viral replication.

**FIG 3:**
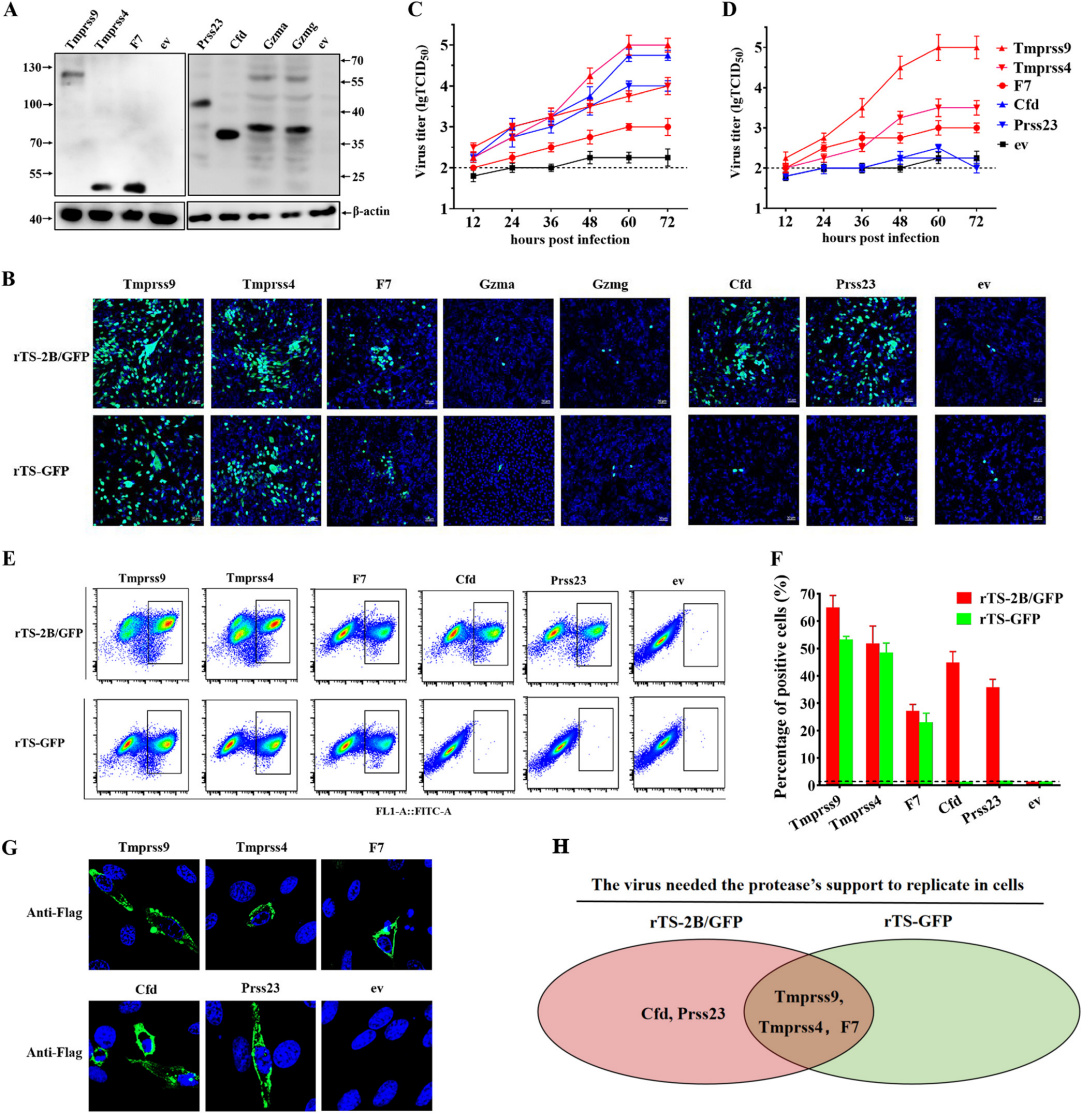
Identification of trypsin-like proteases activating NDV in DF1 cells. (A) Western blot detection of proteases transiently expressed in DF1 cells. DF1 cells were transfected with the plasmids expressing Tmprss9, Tmprss4, F7, Prss23, Cfd, Gzma, and Gzmg, respectively. The empty vector (ev) served as a control. Cells were lysed at 48 h posttransfection and then analyzed for the expression of proteases using mouse serum against Flag. (B) Fluorescence microscopy analysis of GFP expression in rNDV-GFP-infected DF1 cells (MOI, 0.001) transiently expressing the indicated proteases at 48 hpi. (C and D) DF1 cells transiently expressing the indicated proteases were infected with rTS-2B/GFP (C) and rTS-GFP (D) (MOI, 0.1). Media from infected cells were collected at the indicated time points, and viral titers were determined by TCID_50_ titration in BHK-21 cells. (E and F) Flow cytometry analysis of GFP-positive cells, which transiently expressed proteases and which were infected with rNDV-GFPs. The dots representing GFP-positive cells are included in black boxes (E), and the percentages of GFP-positive cells were calculated (F). (G) DF1 cells were transfected with plasmids expressing Tmprss9, Tmprss4, F7, Cfd, and Prss23, respectively. Cells were detected by immunofluorescence for protease expression using anti-Flag antibody at 48 h posttransfection. Then, the localization of proteases in DF1 cells was observed using a confocal microscope. (H) Venn diagram showing two groups of NDV-activating proteases. The red and green groups include proteases that activated rTS-2B/GFP and rTS-GFP, respectively.

Whether the seven candidate proteases (F7, Gzma, Gzmg, Tmprss4, Tmprss9, Cfd, and Prss23) were able to activate NDV in DF1 cells was then investigated by using the transient expression assay. At 48 h posttransfection, the seven candidate proteases were found to be expressed efficiently in DF1 cells (Fig. 3A). Compared with the empty vector (ev) control, the greatly increased GFP green signals were observed from the rTS-2B/GFP-infected cells transiently expressing five proteases (Tmprss9, Tmprss4, F7, Cfd, and Prss23) and from the rTS-GFP-infected cells transiently expressing three proteases (Tmprss9, Tmprss4, and F7) (Fig. 3B). The growth kinetics of NDV in DF1 cells transiently expressing these proteases were determined. At 72 h post infection (hpi), the different levels of rTS-2B/GFP titers from the cells expressing proteases were observed in the order of Tmprss9 > Cfd > Prss23 = Tmprss4 > F7 > ev (Fig. 3C), and those of rTS-GFP titers were observed in the order of Tmprss9 > Tmprss4 > F7 > ev = Cfd = Prss23 (Fig. 3D). Similar results were obtained by quantitatively analyzing the GFP-positive cells using a flow cytometry assay (Fig. 3E and F). When analyzed using confocal microscopy, the identified proteases transiently expressed in DF1 cells were found to be located near the cellular membrane (Fig. 3G). Therefore, our results showed that five trypsin-like serine proteases (Tmprss9, Tmprss4, F7, Cfd, and Prss23) expressed in chicken embryos could activate NDV with 2B-FCS, and three of them (Tmprss9, Tmprss4, and F7) could activate NDV with 3B-FCS (Fig. 3H).

### Cleavage of F proteins by trypsin-like serine proteases *in vivo* and *in vitro*.

**FIG 4:**
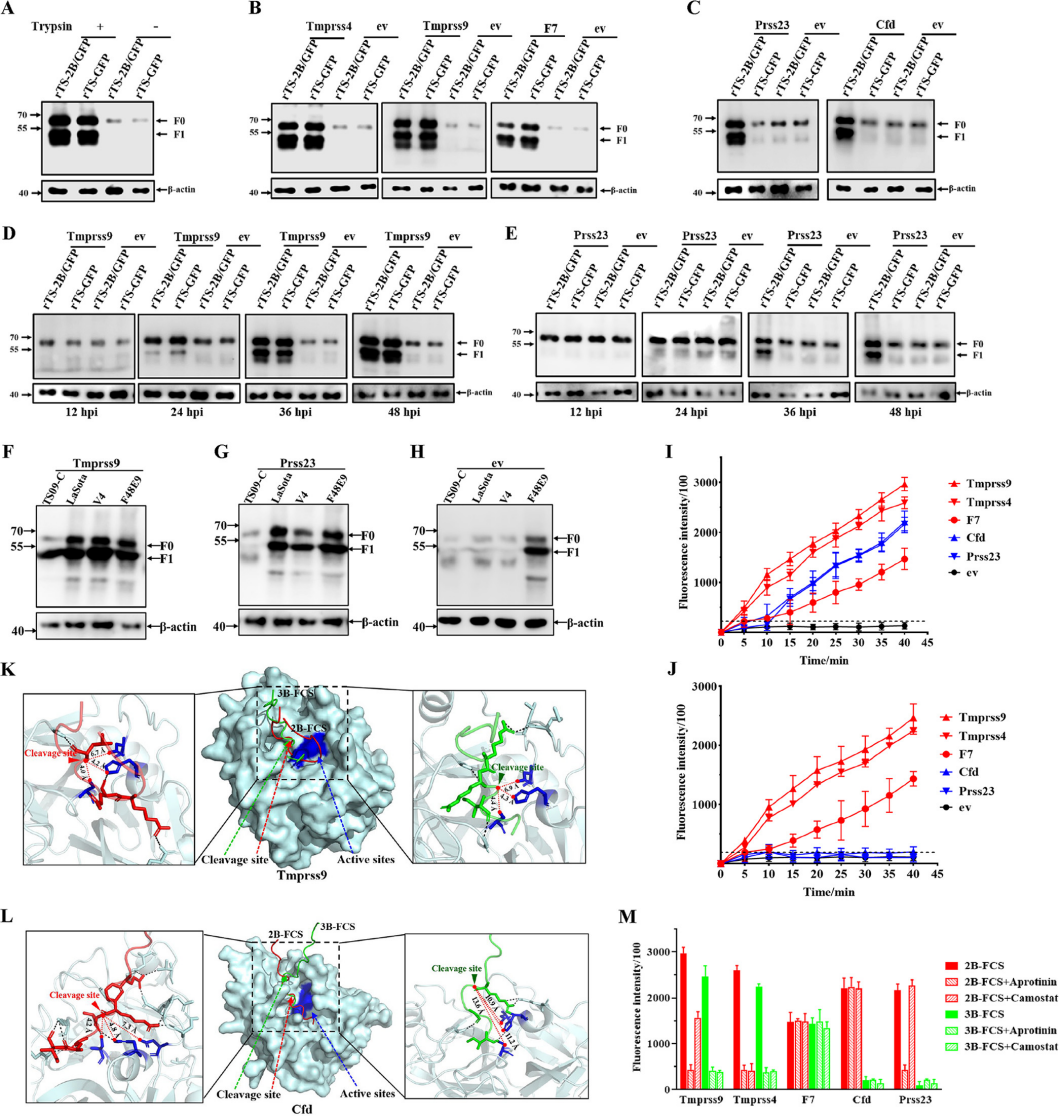
Cleavage of NDV F proteins by trypsin-like proteases *in vivo* and *in vitro*. (A) DF1 cells infected by the indicated rNDVs with or without trypsin were harvested at 48 hpi. Western blotting was performed with mouse serum against F protein. (B and C) Western blot detection of F proteins in rNDV-infected cells transiently expressing proteases Tmprss9, Tmprss4, and F7 (B) and Prss23 and Cfd (C) at 48 hpi. (D and E) Western blot detection of F proteins in rNDV-infected cells transiently expressing Tmprss9 (D) and Prss23 (E) at the indicated time points postinfection. (F, G, and H) DF1 cells transiently expressing Tmprss9 (F), Prss23 (G), and empty vector (H) were infected with the indicated NDV isolates. Cells were lysed at 48 hpi and analyzed for the expression of F protein by a Western blot assay. (I and J) The lysates of cells transiently expressing the indicated proteases were incubated *in vitro* with the synthesized peptides 2B-FCS (I) and 3B-FCS (J) labeled with Edans at 25°C. Then, the cleavage of FCS by proteases was measured by using a fluorescent microplate reader at the indicated time points postincubation. (K and L) Surface and cartoon representation showing the detailed amino acid interactions of proteases Tmprss9 (K) and Cfd (L) with different FCSs. The predicted structures of the complexes of the proteases and FCS were constructed using Alphafold2-multimer in ColabFold. The graph in the middle shows the interaction of the protease (light blue surface) with the target cleavage site of 2B-FCS (red line) and 3B-FCS (green line). The active sites of proteases are indicated in dark blue. Detailed structural views of the interactions of protease with 2B-FCS and 3B-FCS at the catalytic sites of protease are presented on the left and right sides of the graph, respectively. The distances between the FCS cleavage sites and protease active sites are indicated by a red dotted line. (M) The lysates of cells transiently expressing the indicated proteases were incubated with Edans-labeled FCSs, with or without the protease inhibitors aprotinin and camostat. FCS cleavage was then measured at 40 min postincubation.

The FCS cleavage ability of five identified proteases was assessed. As expected, the F proteins of rTS-2B/GFP and rTS-GFP were not cleaved by the endogenous proteases expressed in DF1 cells, and only the precursor protein F0 was detected, while trypsin supported the cleavage of F0 into F1 and F2 (the latter was not detected by the antibody) (Fig. 4A). The proteases Tmprss9, Tmprss4, and F7 could cleave the F proteins from both rTS-2B/GFP and rTS-GFP (Fig. 4B), while Prss23 and Cfd supported only the cleavage of F protein of rTS-2B/GFP, not that of rTS-GFP (Fig. 4C). As the infection progressed, an increase in F protein cleavage was observed in the rTS-2B/GFP- and rTS-GFP-infected cells expressing Tmprss9 (Fig. 4D). However, in the cells expressing Prss23, only an increase in F cleavage of rTS-2B/GFP was observed (Fig. 4E). Furthermore, the F proteins with 2B-FCS from NDV isolates LaSota and V4 could be cleaved by both Tmprss9 and Prss23 and the F protein with 3B-FCS from isolate TS09-C could be cleaved only by Tmprss9, while the F protein with FCS containing four basic amino acids from isolate F48E9 could be cleaved by the endogenous proteases expressed in DF1 cells (Fig. 4F to H). These data demonstrated that all five identified proteases could cleave the F protein with 2B-FCS and that three of them (Tmprss9, Tmprss4, and F7) could cleave the F protein with 3B-FCS.

Next, the cleavage of F protein by the identified proteases was determined *in vitro*. The lysates of DF1 cells transiently expressing these proteases (Fig. 3A) were incubated with the synthesized FCS peptides labeled with the fluorogenic substrate Edans. Compared with the ev control, all the lysates from cells expressing proteases could cleave the 2B-FCS peptide (Fig. 4I), while only three proteases (Tmprss9, Tmprss4, and F7) could cleave the 3B-FCS peptide (Fig. 4J). According to the predicted structures of complexes of proteases and FCS, the protease active sites of Tmprss9 were very close to the cleavage sites of 2B-FCS and 3B-FCS, with the average distances being 5.0 and 5.2 Å, respectively (Fig. 4K). For the protease Cfd, the protease active sites were also very close to the cleavage site of 2B-FCS but far from that of 3B-FCS, with the average distances being 5.4 and 11.9 Å, respectively (Fig. 4L). The results indicated that the protease Cfd was not able to cleave the 3B-FCS due to the far distances between the protease active sites and cleavage site of 3B-FCS. Furthermore, two serine protease inhibitors, aprotinin and camostat, were selected and tested for their inhibitory effects on FCS cleavage by these proteases. As shown in Fig. 4M, aprotinin was the inhibitor of proteases Tmprss9, Tmprss4, and Prss23 and camostat was the inhibitor of Tmprss9 and Tmprss4.

### Biological characteristics of recombinant NDV expressing trypsin-like serine protease.

**FIG 5:**
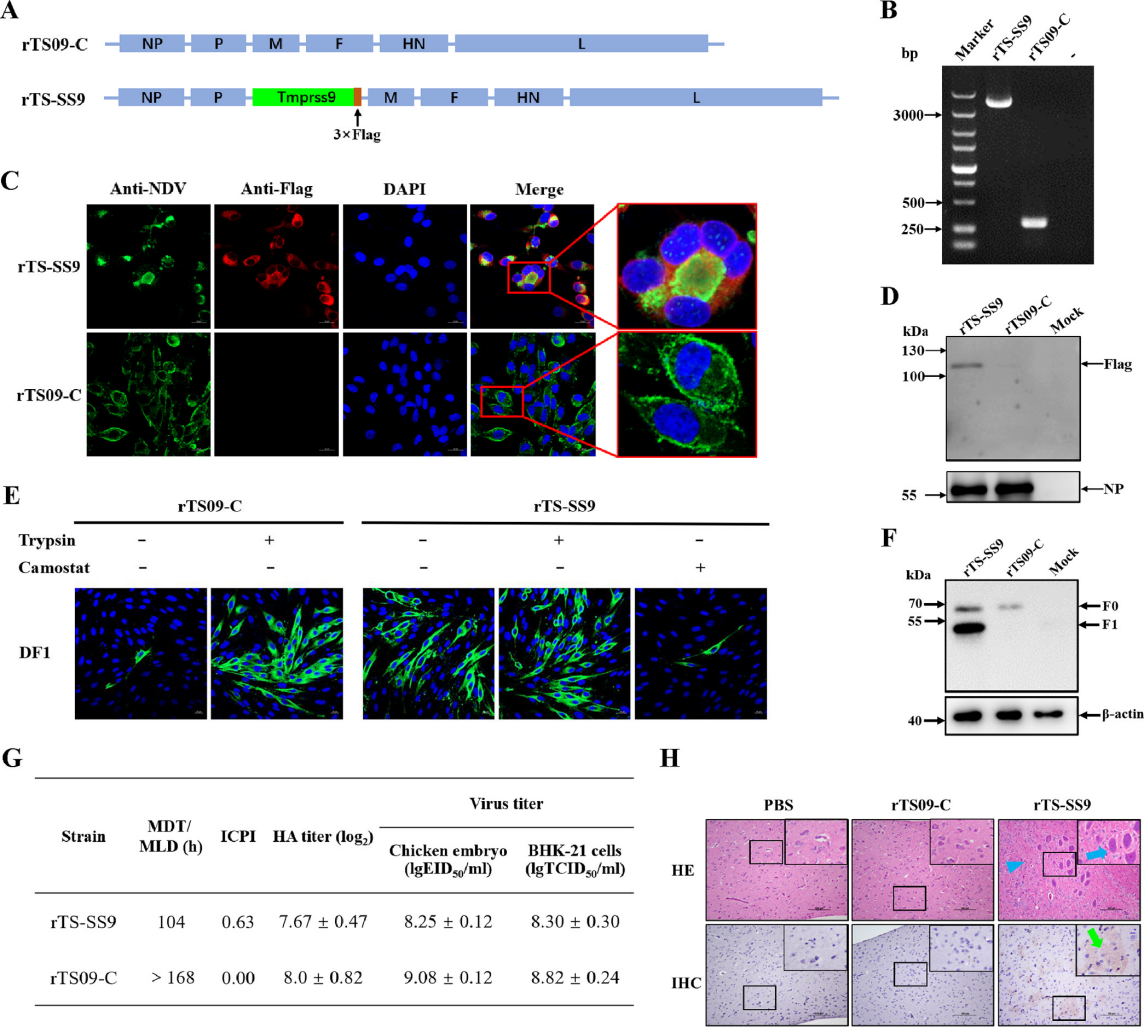
Construction and biological characteristics of recombinant NDV expressing Tmprss9. (A) Schematic presentation of the genomes of rTS09-C and rTS-SS9. (B) Proper insertion of the Tmprss9 gene in rTS-SS9 was confirmed by using a pair of primers (NDV F and NDV R) spanning the Tmprss9 gene insertion region. (C) DF1 cells were infected with rTS-SS9 and rTS09-C (MOI, 0.1) with the addition of TPCK-trypsin. At 48 hpi, cells were subjected to immunofluorescence staining using anti-NDV and anti-Flag antibodies. Cells were analyzed by a confocal microscope. (D) DF1 cells infected with rNDVs (MOI, 0.1) with TPCK-trypsin were lysed at 48 hpi and analyzed for the expression of Tmprss9 and NP proteins by Western blotting using anti-Flag and anti-NDV antibodies, respectively. (E) DF1 cells were infected with rNDVs (MOI, 0.1) with or without trypsin/camostat, and at 48 hpi, cells were subjected to immunofluorescence staining using anti-NDV antibody. (F) Western blot detection of F proteins in DF1 cells infected by rNDVs without TPCK-trypsin, using anti-F antibody. (G) Pathogenicity of rNDVs in chickens and their growth titers in chicken embryos and BHK-21 cells. (H) Brain tissues of chickens that survived rNDV ICPI tests were collected and then subjected to hematoxylin-eosin staining for histopathological analysis and to IHC staining for NDV detection. The blue arrows indicate neuronal swelling, triangles indicate nuclear shrinkage, and green arrows indicate NDV-positive signals.

Next, we investigated whether the expression of proteases affected other biological characteristics of NDV. The Tmprss9 gene was inserted between the P and M genes of the infectious clone of NDV strain TS09-C to generate recombinant NDV expressing Tmprss9, and this strain was named rTS-SS9 (Fig. 5A). The presence of the inserted Tmprss9 gene in strain rTS-SS9 was confirmed by reverse transcriptase PCR (RT-PCR) (Fig. 5B) and sequencing. With the addition of trypsin, both the Tmprss9 and NP proteins were expressed efficiently in the rTS-SS9-infected cells (Fig. 5C and D). Thus, rTS-SS9 with 3B-FCS was constructed successfully and could effectively express Tmprss9 in the infected cells.

The biological characteristics of rTS-SS9 were then evaluated. Without the addition of trypsin, rTS-SS9 could replicate in DF1 cells with the support of Tmprss9 expressed by the virus itself. The addition of the Tmprss9 inhibitor camostat into DF1 cells greatly inhibited the replication of rTS-SS9 (Fig. 5E). By using Western blotting, cleavage of the F protein by Tmprss9 expressed by the virus itself was observed (Fig. 5F). The pathogenicity of rTS-SS9 was greatly increased, with a mean death time of minimal death dose (MDT/MLD) value of 104 h and an intracerebral pathogenicity index (ICPI) value of 0.63, compared with those of rTS09-C strain (MDT/MLD > 168 h, ICPI = 0.00). The growth titers of rTS-SS9 in chicken embryos and BHK-21 cells were slightly lower than those of rTS09-C (Fig. 5G). The brain tissues of birds that survived the NDV ICPI tests were collected and examined by histopathology and an immunohistochemistry (IHC) assay. Moderate histopathological lesions, such as neuronal swelling and nuclear shrinkage, were observed in the brains of the rTS-SS9 group, while no lesions were observed in the rTS09-C group. Many more NDV-positive signals were observed in the brains from the rTS-SS9 group than in those of the rTS09-C group (Fig. 5H). Our results suggested that the insertion and expression of Tmprss9 in NDV with 3B-FCS supported the cleavage of viral F protein and the replication of virus in DF1 cells, which increased the pathogenicity of virus in chickens.

### Effect of trypsin-like serine protease inhibitors on the activation of NDV in primary chicken embryo cells.

**FIG 6:**
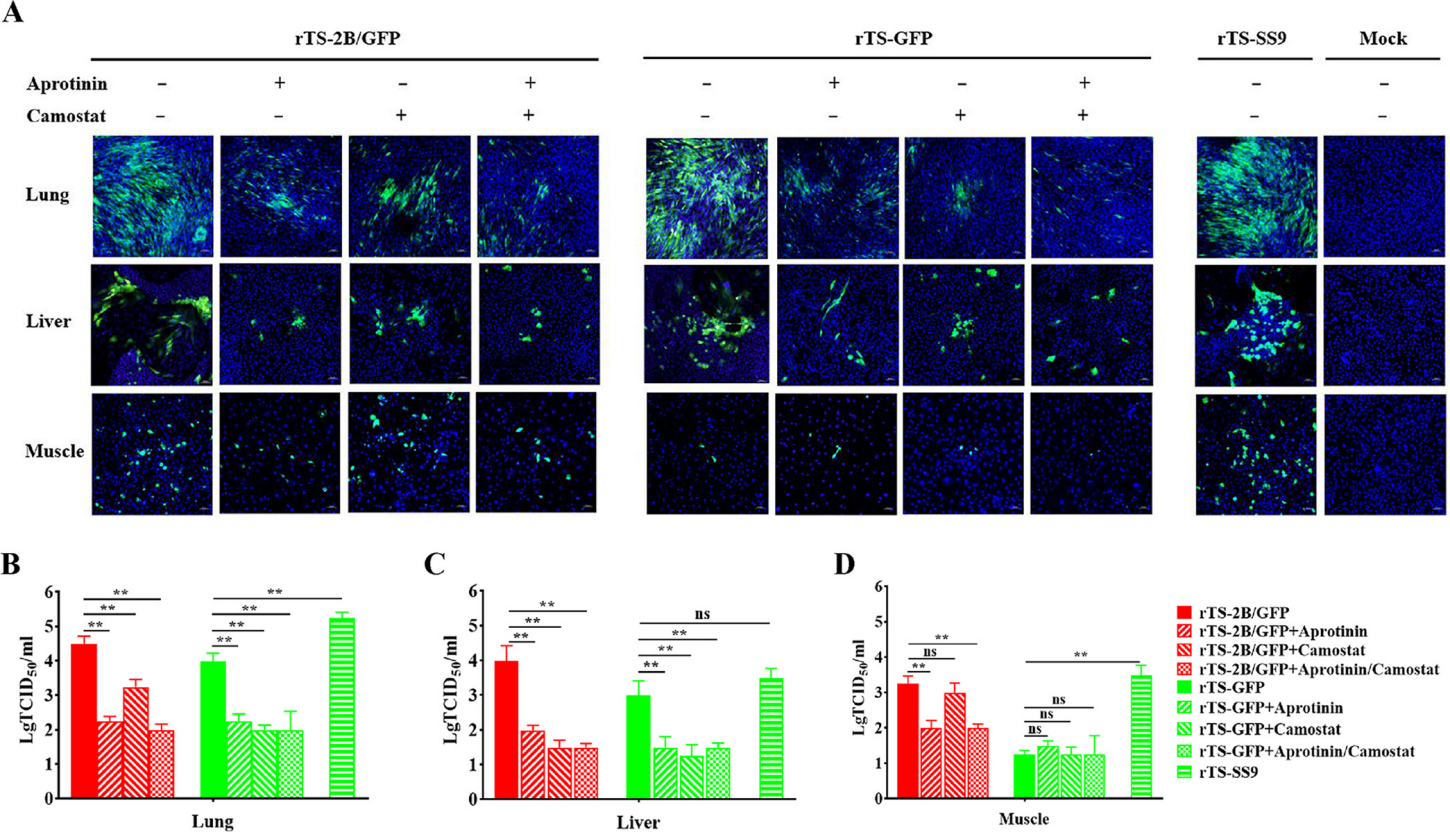
Effect of protease inhibitors on NDV activation in primary cells. (A) Fluorescence microscopy analysis of GFP expression in chicken embryonic primary cells infected by rTS-2B/GFP and rTS-GFP (MOI, 0.001) with or without aprotinin/camostat at 48 hpi. The rTS-SS9-infected cells were immunofluorescence stained by using anti-NDV antibody at 48 hpi and analyzed using fluorescence microscopy. (B to D) Viral titers in chicken embryonic primary lung (B), liver (C), and muscle (D) cells infected by rTS-2B/GFP, rTS-GFP, and rTS-SS9 at 36 hpi, with or without the indicated protease inhibitors. Media from infected cells were harvested at 36 hpi, and viral titers were determined by TCID_50_ titration in BHK-21 cells. Statistical significance in the viral titer was determined with two-tailed *t* tests (ns, *P* > 0.05; *, 0.01 < *P* < 0.05; **, *P* < 0.01).

The effect of the identified proteases on viral tissue tropism was further investigated. In primary lung and liver cells, the replications of rTS-2B/GFP and rTS-GFP were greatly inhibited by the addition of aprotinin (inhibitor of Tmprss9, Tmprss4, and Prss23) and camostat (inhibitor of Tmprss9 and Tmprss4), and the replication titers of rTS-2B/GFP and rTS-GFP were significantly higher than those with aprotinin and camostat (Fig. 6A to C). In primary muscle cells, the replication of rTS-2B/GFP was inhibited by aprotinin but not by camostat (Fig. 6A and D). Thus, in the lung and liver cells, the FCS cleavage activity of Tmprss9, Tmprss4, and Prss23 was inhibited by aprotinin and camostat, leading to the failure of recombinant NDVs (rNDVs) with both 2B-FCS and 3B-FCS to be efficiently activated and subsequently replicate. In the muscle cells, the FCS cleavage activity of protease Prss23 could be inhibited only by aprotinin, and thus, the rNDV with 2B-FCS could also not be efficiently activated or replicate with the addition of aprotinin. In contrast, the rTS-SS9 with 3B-FCS could replicate efficiently in lung, liver, and muscle cells with the support of Tmprss9 expressed by the virus itself (Fig. 6A and D). These results demonstrated that the activation and replication of NDV in primary chicken embryo cells strictly depended on the FCS cleavage by the identified proteases expressed in these cells, which could be greatly affected by the protease inhibitors.

### Effect of trypsin-like serine proteases on the pathogenicity of NDV in chicken embryos.

**FIG 7:**
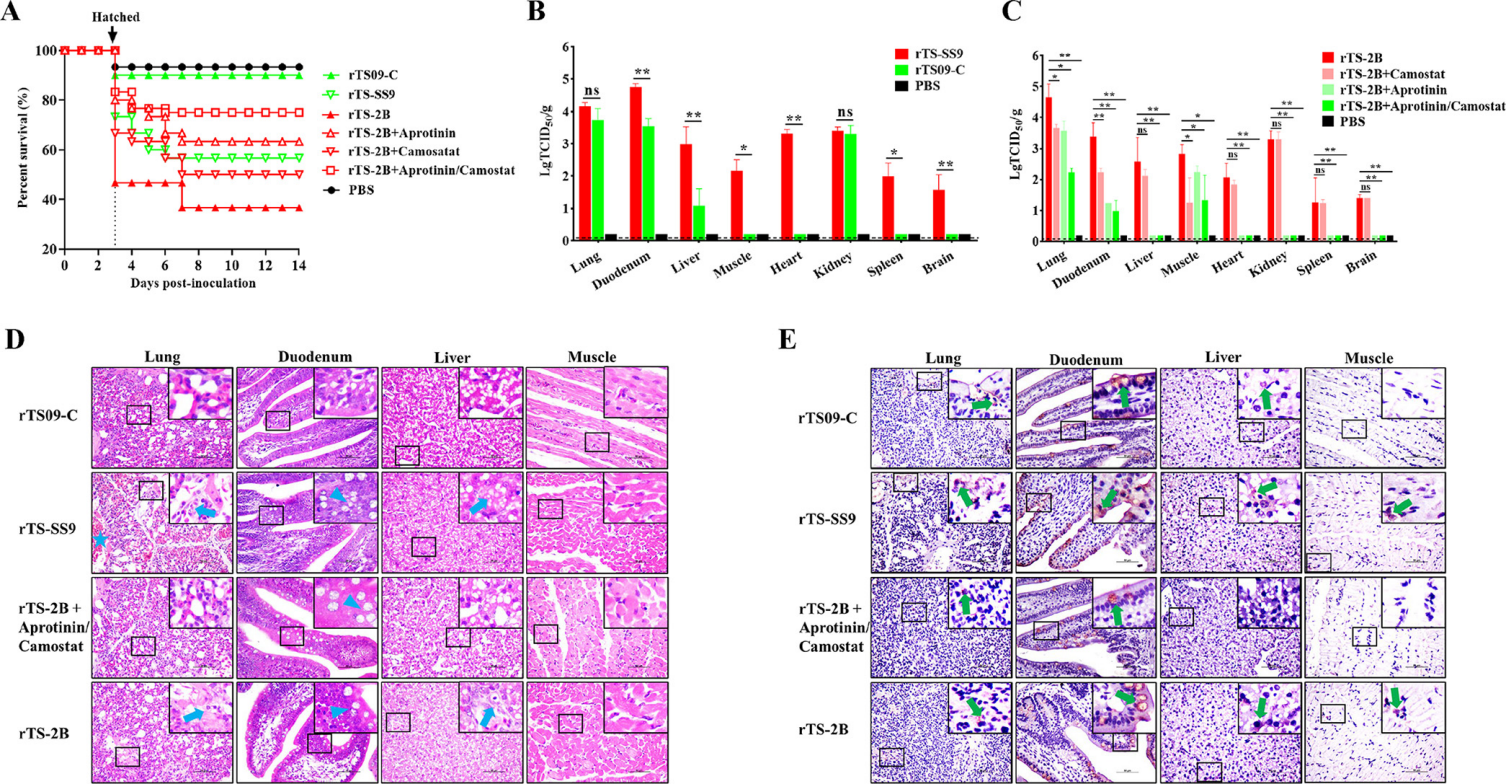
Effect of proteases on the pathogenicity of NDV in chicken embryos. (A) Percentages of survival of chicken embryos vaccinated *in ovo* with recombinant NDV strains rTS09-C, rTS-SS9, and rTS-2B with or without the addition of protease inhibitors. (B) Viral titers in eight kinds of tissues collected from chickens vaccinated *in ovo* with rTS09-C and rTS-SS9 at 1 dpi. (C) Viral titers in eight kinds of tissues collected from chickens inoculated *in ovo* with rTS-2B with or without the indicated protease inhibitors at 1 dpi. The viral titer of each sample was determined in BHK-21 cells. Statistical significance of the viral titer was determined by two-tailed *t* tests (ns, *P* > 0.05; *, 0.01 < *P* < 0.05; **, *P* < 0.01). (D) Histopathological analysis of tissue samples from chickens vaccinated *in ovo* with rTS09-C, rTS-SS9, rTS-2B, and rTS-2B with the addition of aprotinin and camostat at 1 dpi. The lesions are indicated by different symbols (arrows, cell necrosis; triangles, goblet cell proliferation; stars, hemorrhage). (E) IHC detection of rNDVs in tissue samples from *in ovo*-vaccinated birds at 1 dpi. The green arrows indicate NDV-positive signals.

To evaluate the effect of the identified proteases on viral pathogenicity in chicken embryos, the pathogenicity of strain rTS-SS9 in 18-day-old chicken embryos was first tested. As shown in Fig. 7A, the hatching rate and survival rate of the rTS-SS9 group at 14 dpi were 73.3% and 56.7%, respectively, which are much lower than those of the parental rTS09-C group (93.3%). The tissue tropism of rTS-SS9 was then determined. Strain rTS-SS9 could replicate in all eight tissues, while rTS09-C could replicate only in some of the tissues (4/8). The titers of rTS-SS9 in duodenum and liver tissues were significantly higher than those of rTS09-C (Fig. 7B). Results demonstrated that protease Tmprss9 expressed by NDV could greatly increase viral replication, tissue tropism, and subsequent pathogenicity in chicken embryos.

Next, the effect of proteases on the pathogenicity of rTS-2B in chicken embryos was investigated by using a protease inhibitor. The hatching rates and survival rates of protease inhibitor groups were much higher than those of the control group (Fig. 7A). The tissue tropisms of virus were then determined. With the addition of camostat, rTS-2B could still replicate in all eight tissues, but the viral replication titers in four tissues from this group were significantly lower than those of the control group. With the addition of aprotinin, rTS-2B could replicate in only three tissues (lung, duodenum, and muscle), and the replication titers in these tissues were significantly lower than those of the control group and were not even detected in some tissues (Fig. 7C). Compared with the rTS-2B control group, fewer pathological lesions (only goblet cell proliferation in duodenum) and greatly decreased NDV-positive signals were observed in the inhibitor group (rTS-2B plus aprotinin or camostat) (Fig. 7D and E). Furthermore, neither the addition of protease inhibitors nor the expression of protease significantly affected the NDV-specific antibody response of birds induced by recombinant virus (see Fig. S3 in the supplemental material). The pathogenicity of strain LaSota in chicken embryos was also decreased with the addition of protease inhibitors (Fig. S4). These data demonstrated that the identified trypsin-like serine proteases could affect the pathogenicity of NDV in chicken embryos.

## DISCUSSION

**FIG 8:**
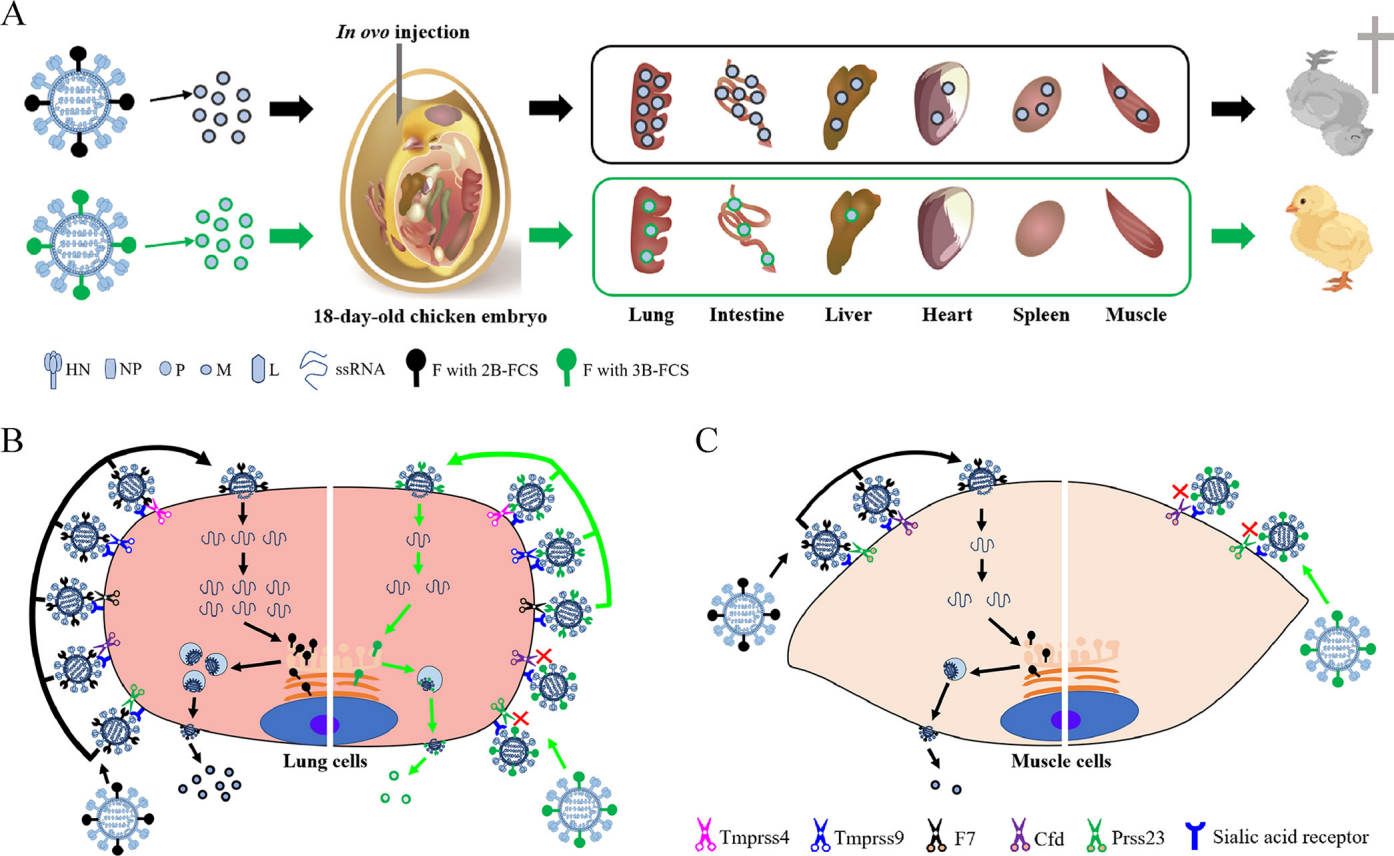
Proposed mechanism for the pathogenicity of NDV in chicken embryos. (A) When vaccinated *in ovo* into chicken embryos, NDV with 2B-FCS could replicate efficiently in most of the chicken embryo tissues, including lung, intestine, liver, heart, spleen, and muscle, leading to broad tissue tropism and the subsequent death of birds, while NDV with 3B-FCS could replicate only inefficiently in fewer chicken embryo tissues, including lung, intestine, and liver, leading to limited tissue tropism and the subsequent survival of birds. (B) In lung cells, five NDV-activating proteases were identified, including Tmprss9, Tmprss4, F7, Cfd, and Prss23. All of them could cleave the F protein with 2B-FCS into subunits F1 and F2. Together with HN protein, the more-cleaved F proteins efficiently initiate the fusion of virion envelope with the host cell plasma membrane and high-level replication of virus, while the F protein with 3B-FCS could be cleaved only by three proteases (Tmprss9, Tmprss4, and F7). The less-cleaved F proteins could inefficiently initiate the fusion of membranes and low-level viral replication in lung cells. (C) In muscle cells, only two NDV-activating proteases, Cfd and Prss23, were identified. Both of them could cleave the F protein with 2B-FCS, and the less-cleaved F proteins inefficiently initiated membrane fusion and subsequent low-level replication of virus. However, none of them could cleave the F protein with 3B-FCS. Thus, the uncleaved F proteins could not initiate membrane fusion and the subsequent viral replication in muscle cells.

Our previous study reported that the pathogenicity of NDV in chicken embryos was determined by the FCS of the virus. Here, five embryonic chicken proteases cleaving the FCS of NDV were further identified, and their roles in viral activation, tissue tropism, and subsequent pathogenicity in chicken embryos were elucidated. Being mixed with the protease inhibitors, the NDV displayed greatly decreased pathogenicity in chicken embryos. Thus, a model was proposed specifying that the identified host proteases should be “the key determinant” for NDV pathogenicity in chicken embryos (Fig. 8).

When *in ovo* vaccinated into chicken embryos, NDV with 2B-FCS could efficiently replicate in most chicken embryo tissues, such as lung, intestine, liver, heart, spleen, and muscle, which led to broad tissue tropism and the subsequent death of birds, while NDV with 3B-FCS could replicate only inefficiently in a few chicken embryo tissues, including lung, intestine, and liver, which led to limited tissue tropism and the subsequent survival of birds (Fig. 8A). Two representative chicken embryo tissue cells, lung and muscle cells, were selected to elucidate the molecular mechanism for viral pathogenicity in chicken embryos. In lung cells, the F protein with 2B-FCS could be cleaved efficiently by five kinds of proteases expressed in lung cells and then the virus could efficiently initiate the fusion of viral envelope with the host cell plasma membrane and subsequent high-level replication of virus, while the F protein with 3B-FCS could be cleaved by only three proteases. The less-cleaved F proteins inefficiently initiated the fusion of membranes and a low level of viral replication in lung cells (Fig. 8B). In muscle cells, only two NDV-activating proteases were identified. Both of them could cleave the F protein with 2B-FCS, and then the less-cleaved F proteins initiated a low level of virus replication. However, none of them could cleave the F protein with 3B-FCS, which hampered the replication of virus in muscle cells (Fig. 8C). Our findings highlighted the importance of embryonic chicken proteases in the biological characterization of NDV.

Type II transmembrane serine proteases (TTSPs), a family of trypsin-like membrane-anchored serine proteases (22), have been confirmed to activate a wide range of viruses, such as severe acute respiratory syndrome virus (SARS), Middle East respiratory syndrome coronaviruses, human metapneumoviruses, and influenza viruses (23–25). The human TTSPs, such as Tmprss2, Tmprss4, and Tmprss9, playing a crucial role in the entry process for S protein priming, were essential for viral infectivity (26, 27). The protease Tmprss2 was identified as the first human protease activating influenza A virus (IAV) hemagglutinin (HA) with a monobasic cleavage site *in vitro* (28). Thereafter, a number of TTSPs have been shown to activate IAV HA sites and more recently influenza B virus HA with a monobasic cleavage site (29–32). In addition to TTSPs, the cathepsin family of endosomal proteases was required for proteolytic processing of several viruses during entry into host cells (33, 34). Here, we reported five NDV-activating proteases expressed in chicken embryos. Among them, Prss23 (serine protease 23), Cfd (complement factor D, also known as adipsin), and F7 (coagulation factor VII) were identified first as virus-activating proteases.

Generally, the virulence of NDV was positively correlated with the number of basic residues in the FCS (35). The NDV strains containing an FCS with four or five basic amino acids were virulent strains, and viruses containing 2B-FCS were avirulent strains. However, the virulence of TS09-C virus with 3B-FCS was lower than that of NDV with 2B-FCS, which was inconsistent with previous reports (17, 19, 36). Here, it was demonstrated that NDV isolates LaSota and V4 containing 2B-FCS could be activated by five embryonic chicken proteases, Tmprss9, Tmprss4, F7, Cfd, and Prss23, while NDV isolate TS09-C containing 3B-FCS could be activated by only three of them (Tmprss9, Tmprss4, and F7). It was assumed that the structural stability of the complex of FCS and protease, and the distances between the cleavage site of FCS and the active sites of protease in the complex, might account for the specificity of the FCS cleavage activity of cellular protease and subsequent viral pathogenicity.

The molecular mechanism for the attenuation of NDV with 2B-FCS in hatched birds has been well known. However, little has been known about the mechanism for the high pathogenicity of this virus in chicken embryos. Five NDV-activating proteases expressed in chicken embryos were identified in this study, and all of them could activate NDV with 2B-FCS. Among them, Cfd and Prss23 were found to be expressed in most chicken embryo tissues, and thus these two proteases were mainly responsible for the viral systemic replication, broad tissue tropism, and high pathogenicity in chicken embryos. Therefore, it is suggested that the differences between hatched birds and chicken embryos in the expression and distribution of cellular proteases determined the different pathogenicities of NDV with 2B-FCS in them.

Many viruses, such as influenza virus, SARS-CoV-2, infectious bronchitis virus, and NDV, require host proteases to cleave their attachment or fusion proteins to activate the infectivity of virus via the protease-mediated entry mechanism (37–40). The protease inhibitors targeting host proteases that are critical to viral replication have been employed as antiviral drugs for viruses (41, 42). Aprotinin and camostat have been actively studied as anti-influenza virus and anti-coronavirus drugs that inhibit transmembrane serine protease 2 (Tmprss2), which is involved in the cleavage of HA and S proteins, and the activation of viruses (43–46). Here, we further demonstrated that aprotinin was the inhibitor of proteases Tmprss9, Tmprss4, and F7 and that camostat was the inhibitor of Tmprss9 and Tmprss4. When tested in chicken embryos, both of these protease inhibitors could greatly decrease the replication, tissue tropism, and pathogenicity of NDV with 2B-FCS, suggesting a novel strategy for the development of *in ovo* vaccines.

The hatchability efficiency of immunized chicken embryos was a crucial factor for the evaluation of *in ovo* vaccine. For NDV, most of the vaccine strains used safely and efficiently for hatched birds were not suitable for chicken embryos due to their high pathogenicity. Unlike the previously described methods, new methods have been suggested in this study for use in decreasing the pathogenicity of vaccines in chicken embryos. First, the sensitivity of viral F protein to cellular proteases could be decreased by site-directed mutation in its FCS. Second, by inserting the genes of protease inhibitors into the genome of NDV, the viral replication level and tissue tropism could be limited. Third, when mixed with the protease inhibitor, the vaccine could display decreased replication levels and limited tissue tropism in chicken embryos.

In conclusion, five proteases, Tmprss9, Tmprss4, F7, Cfd, and Prss23, were identified as NDV F-cleaving proteases in chicken embryos. The activation of NDVs with 2B-FCS and 3B-FCS was triggered by different but overlapping sets of proteases in chicken embryos. The Cfd and Prss23 expressed in most chicken embryo tissue cells did not cleave 3B-FCS but rather 2B-FCS, and thus their contributions to different pathogenicities of NDV with 2B-FCS and 3B-FCS in chicken embryos were indicated. Our findings extend the understanding of the molecular mechanism of NDV pathogenicity in chicken embryos and provide a molecular basis for the rational design of *in ovo* vaccines ensuring uniform and effective vaccine delivery and earlier induction of immune protection by the time of hatching.

## MATERIALS AND METHODS

### Animals and ethics statement.

SPF Leghorn chicken embryos were purchased from Merial-Vital, Beijing, China. The chicken embryos were hatched in a contained environment at 37.5°C and ~50 to 60% humidity.

All the animal experiments were approved (permit number 17/2019) and supervised by the Institutional Animal Care and Use Committee of the Hubei Academy of Agriculture Sciences, Wuhan, China.

### Cells, viruses, and reagents.

DF1 and BHK-21 cells were cultured in Dulbecco’s modified Eagle’s medium (DMEM) supplemented with 10% fetal bovine serum (FBS) at 37°C with 5% CO_2_. The chicken embryo primary cells, including lung, liver, and muscle cells, were isolated from 18-day-old SPF chicken embryos as described previously (47). Briefly, these tissues were recovered and placed in ice-cold Hanks’ balanced salt solution (HBSS), cleaned from fat and external mucus, and cut into pieces of 1 to 2 mm. The tissue pieces were washed in ice-cold HBSS and then digested for 15 min at 37°C with collagenase (Sigma-Aldrich). These digested tissues were centrifuged at 800 × *g* for 3 min and washed with PBS. The pellet was resuspended and filtered with a 40-μm-pore-size nylon filter, and thus single cells were obtained. The isolated cells were then cultured in DMEM with 10% FBS, 100 U/mL penicillin (HyClone), and 100 g/mL streptomycin (HyClone) at 37°C with 5% CO_2_.

The four NDV isolates, TS09-C, V4, LaSota, and F48E9, were propagated in SPF chicken embryos.

TPCK (tosylsulfonyl phenylalanyl chloromethyl ketone)-trypsin was purchased from Sigma-Aldrich (St. Louis, MO, USA) and used for supporting NDV replication in cells at a concentration of 0.2 μg/mL. The protease inhibitors camostat and aprotinin were purchased from MedChemExpress LLC (Shanghai, China) and Merck (Darmstadt, Germany), respectively. The protease inhibitors were used for the inhibition of F protein cleavage by proteases in cells and chicken embryos at concentrations of 2.5 μg/mL and 0.25 mg/egg, respectively.

### Construction and rescue of recombinant viruses.

The recombinant NDV strains rTS09-C, rTS-2B, and rTS-GFP (the green fluorescent protein [GFP] gene was inserted into the P-M gene of rTS09-C virus containing three basic amino acids in the FCS) were constructed and rescued previously (17, 48) and maintained in our laboratory.

For the construction of the full-length cDNA clones of rTS-SS9 and rTS-2B/GFP, strains rTS09-C and rTS-2B were used as the backbone virus, respectively. Briefly, the Tmpress9 gene fragment was amplified from lung tissue of 18-day-old SPF chicken embryos. Tmprss9 and GFP genes were then inserted into the P-M gene of TS09-C and TS-2B, respectively, using an In-Fusion PCR cloning kit (TaKaRa), to generate plasmids pTS-SS9 and pTS-2B/GFP.

To rescue the recombinant NDV, the constructed full-length cDNA clone (pTS-SS9 or pTS-2B/GFP) was cotransfected into MVA-T7-infected BHK-21 cells, with the NP, P, and L supporting plasmids, by using Lipofectamine 3000 (Invitrogen). At 6 h posttransfection, the cells were washed with PBS and cultured in serum-free DMEM, antibiotics, and TPCK-trypsin. The cocultures were incubated for 72 h before inoculation into 10-day-old SPF chicken embryos. At 96 hpi, the allantoic fluids were harvested and the virus was identified by an HA assay and RT-PCR sequencing.

### Virus titration and pathogenicity tests.

The titers of NDV in allantoic fluids, chicken embryo tissues, and cultured cells were determined by using the standard HA assay in 96-well microplates, the 50% egg infectious dose (EID_50_) assay in 10-day-old SPF chicken embryos, and the 50% tissue culture infectious dose (TCID_50_) assay on BHK-21 cells in the presence of TPCK-trypsin. The EID_50_ and TICD_50_ values were calculated using the Reed-Muench method.

The pathogenicity of NDV for chickens was tested by performing the ICPI assay in 1-day-old SPF chickens and the MDT/MLD assay in 10-day-old SPF chicken embryos (2).

### RNA isolation from tissues of chicken embryos.

For the isolation of total RNA from tissues of chicken embryos, 18-day-old chicken embryos were sacrificed, and the lung, liver, and muscle tissues were collected and washed in sterilized PBS. Thirty milligrams of tissue was frozen in liquid nitrogen and homogenized by using a precooled mortar/pestle. The total RNA was isolated from homogenized tissues by using the RNeasy kit (Qiagen, Beijing, China), according to the manufacturer’s protocol. The isolated RNA was stored at −80°C before use.

### RNA-seq and bioinformatic analysis for gene expression profiling.

The quality of total RNA isolated from chicken embryo tissues was assessed with an Agilent 2100 bioanalyzer (Thermo Fisher Scientific, MA, USA), and library preparation was performed with the MGI Easy Universal DNA library prep kit (MGI Tech Co., Shenzen, China) according to the manufacturer's instructions. Sample sequencing was performed on a DNBSEQ-T7 sequencer using mRNA derived from three biological replicates for each tissue. The reads obtained by RNA-seq were aligned with the reference genome by HISAT2 (v2.0.4). Prior to expression profiling, transcripts were prefiltered to those that yielded at least one FPKM (fragment per kilobase per million) value of ≥0.3 and a tag count of ≥50 out of three replicates to exclude insufficiently covered genes from the analysis. Protease genes were filtered based on gene ontology classification and according to enzyme classification (EC) as hydrolases (EC 3.4). Serine protease genes were filtered with reference to EC 3.4.21 classified genes as well as UniProt and MEROPS database searches to filter for proteases with trypsin-like activity. RNA-seq data have been deposited in the NCBI database (accession number PRJNA903649).

### RT-qPCR assay.

The total RNA isolated from chicken embryo tissues was quantitatively analyzed using a NanoDrop 2000 spectrophotometer. Approximately 1 μg of total RNA was reverse transcribed using a two-step Moloney murine leukemia virus (MMLV) RT-PCR kit (GeneMark), and qPCR was performed to determine the expression level of proteases on a Roche LightCycler 96, using SYBR green real-time PCR master mix (GeneMark), according to the manufacturer's instructions. The mRNA expression levels of different proteases are shown as the mean threshold cycle (*C_T_*) values from three replicates.

### Plasmid construction and transfection.

Full-length cDNAs encoding proteases Tmprss9, Tmprss4, F7, Gzma, Gzmg, Cfd, and Prss23 were amplified by RT-PCR from the total RNA isolated from liver tissue or trachea tissue of 18-day-old SPF chicken embryos by using specific primers (available upon request), respectively, and cloned into p3×Flag-CMV (Sigma; E7908). DF1 cells were seeded in 6-well plates and transfected with the constructed plasmids (5.0 μg/well) after 80% confluence was reached by using Lipofectamine 3000 (Invitrogen), according to the manufacturer's instructions.

### Flow cytometry.

DF1 cells cultured in a 6-well plate were transfected with the plasmids expressing proteases. At 24 h posttransfection, cells were infected with rTS-2B/GFP and rTS-GFP at a multiplicity of infection (MOI) of 0.1. At 48 hpi, cells were digested and GFP-positive cell subsets were detected by using a LSR-II flow cytometer, and the acquired data were analyzed by FlowJo v10 software.

### *In vitro* FCS cleavage assay.

The ternary complexes including the FCS, fluorescence signals, and fluorescence quenching were synthesized by Sangon Biotech, Shanghai, China. The ternary complex 3B-FCS was Dabcyl-TSGGGKQRR↓LIGAI-Edans, and the 2B-FCS was Dabcyl-TSGGGKQGR↓LIGAI-Edans. The DF1 cells cultured in a 6-well plate were transfected by protease expression plasmids (5 μg/well) and lysed at 48 h posttransfection. The optimized FCS cleavage reaction was performed in a final volume of 100 μL containing 80 μL of HEPES buffer (20 mM, pH 8.0), 10 μL of cell lysates, and 10 μL of ternary complex (10 μg/mL). After incubation at 25°C for the indicated time, the cleavage reaction solution was detected by using a fluorescent microplate reader at an absorbance at 460 nm.

### Western blotting and immunofluorescence assay.

Cells were washed and lysed with lysis buffer (Beyotime) containing 1 mM phenylmethylsulfonyl fluoride (PMSF). The collected cell lysates were analyzed by Western blotting using the specific chicken anti-NDV (prepared in our laboratory), mouse anti-F (prepared in our laboratory), or mouse anti-Flag (BOSTER) antibodies. For immunofluorescence staining, the cells grown in a 6-well plate were fixed with 4% paraformaldehyde. The fixed cells were blocked in PBS containing 1% bovine serum albumin (BSA) at 25°C for 1 h and incubated with primary antibody (chicken anti-NDV and/or mouse anti-Flag antibodies) for 2 h at 25°C and then with secondary antibody (fluorescein isothiocyanate [FITC]-labeled goat anti-chicken IgG and/or Alexa Fluor 594-labeled goat anti-mouse IgG; Beyotime) for 1 h at 25°C. Cells were imaged under a confocal microscope.

### Structure modeling of the complex of protease and FCS.

The structure of the complex of protease and FCS was molecularly modeled using Alphafold2-multimer via Google Colab (https://colab.research.google.com/github/sokrypton/ColabFold/blob/main/AlphaFold2.ipynb), with the mode of “MMSeqs2 (Uniref + Environmental)” and pair_mode of “unpaired+paired.” Three recycles for all five models were used, and the best prediction according to the multimer score was picked and then visualized by PyMOL version 2.5.4.

### *In ovo* vaccination.

The method of *in ovo* vaccination has been described previously (7). Briefly, 18-day-old SPF chicken embryos were inoculated with one NDV strain at a dose of 10^3.0^ EID_50_/egg with or without the protease inhibitors or an equal volume of PBS via the amniotic route. The vaccinated eggs were hatched in separate incubators. The proportions of inoculated embryos that hatched successfully without assistance and survived to 14 dpi were recorded. At the indicated time points postinoculation, three birds from each group were sacrificed randomly. The 14 kinds of tissues, including lung, trachea, larynx, duodenum, cecum, proventriculus, liver, muscle, heart, kidney, spleen, thymus, bursa fabricius, and brain, were collected from all of the killed chickens, and the NDV titer for each was determined by a TCID_50_ assay. Parts of the lung, duodenum, liver, and muscle tissues were fixed in 4% paraformaldehyde, paraffin embedded, sectioned, and then histopathology stained with hematoxylin-eosin or IHC stained with primary antibody (chicken anti-NDV antibody) and secondary antibody (horseradish peroxidase-labeled goat anti-chicken IgG; Bioss) and analyzed microscopically.

### Statistical analysis.

Data are expressed as means ± standard errors of the means (SEM). Statistical analysis was performed by a paired two-tailed Student *t* test. A *P* value of ≤0.05 was considered significant (*, 0.01 < *P *≤ 0.05; **, *P *≤ 0.01).
