# Supplementary material for: Identification of Embryonic Chicken Proteases Activating Newcastle Disease Virus and Their Roles in the Pathogenicity of Virus Used as In Ovo Vaccine
Source: J Virol. 2023 Apr 12;97(5):e00324-23. doi: 10.1128/jvi.00324-23 (PMC10231145; doi:10.1128/jvi.00324-23)
Supplement: Supplemental file 1 — Fig. S1 to S4. Download jvi.00324-23-s0001.docx, DOCX file, 0.9 MB [file jvi.00324-23-s0001.docx]

**SUPPLEMENTAL FILE 1**


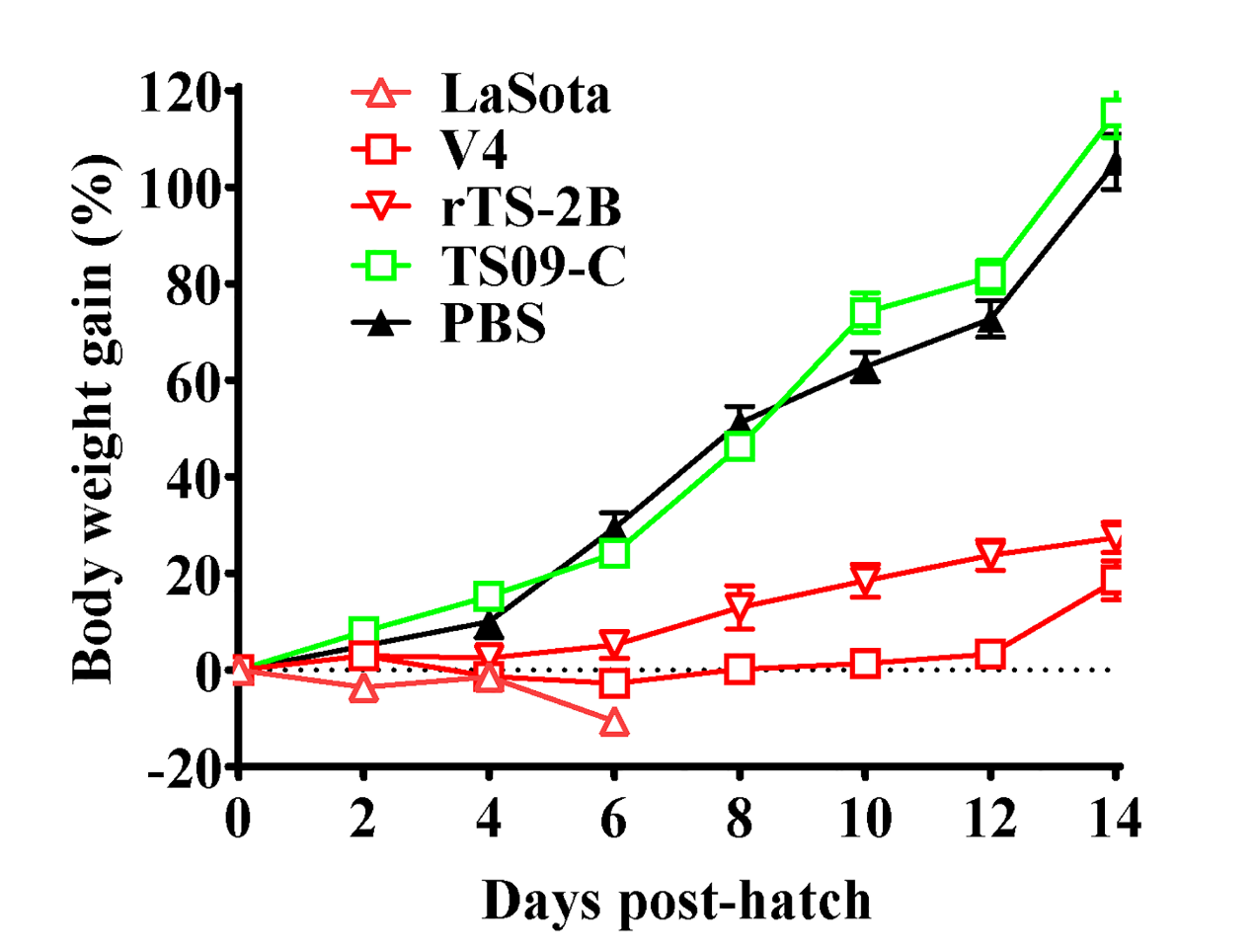


**FIG. S1. Percentage of body weight gain of chicken *in ovo* vaccinated with NDV.** SPF chicken embryos were immunized with different NDV strains or PBS in a 0.1 ml volume (10^3.0^ EID_50_ per egg) via the amniotic route at 18 embryo-days. The birds in each group were weighted at the indicated time points post-hatch. Percentage of body weight gain (%) = (Body weight [the indicated day post-hatch] – body weight [0 day post-hatch]) / body weight [0 day post-hatch] × 100.


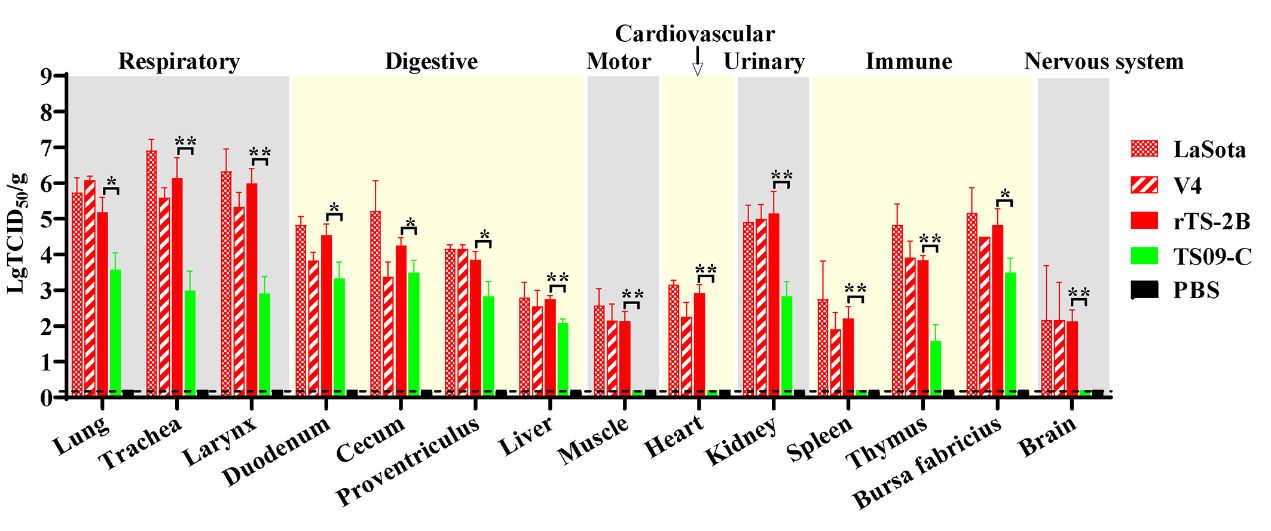


**FIG. S2. Viral replication in the tissues of chickens *in ovo* inoculated with NDV at 5 dpi.** SPF chicken embryos were inoculated with different NDV strains or PBS in a 0.1 ml volume (10^3.0^ EID_50_ per egg) via the amniotic route at 18 embryo-days. Three birds from each group were sacrificed at 5 dpi, then fourteen kinds of tissues were collected. The viral titer of each sample was determined in BHK-21 cells. Statistical significance in the viral titer was determined with two-tailed t tests (ns, *p* > 0.05; *, 0.01 < *p* < 0.05; **, *p* < 0.01).


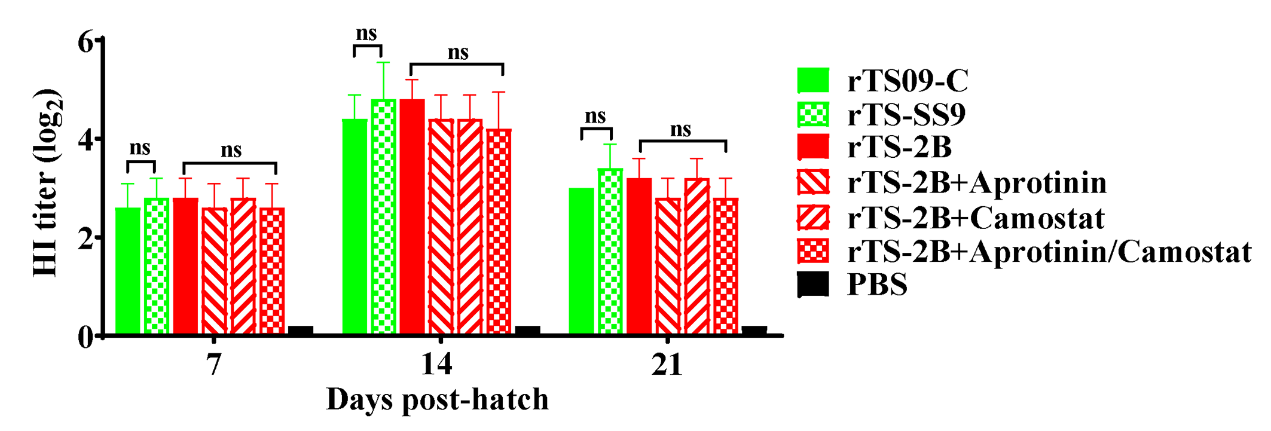


**FIG. S3. NDV-specific antibody responses of chickens *in ovo* vaccinated with NDV.** SPF chicken embryos were *in ovo* inoculated with the indicated NDV strains, with or without the addition of protease inhibitors in a 0.1 ml volume (10^3.0^ EID_50_ per egg) via the amniotic route at 18 embryo-days. Blood sample from five birds in each group were collected at 7, 14, and 21 dph. The NDV antibody was detected by hemagglutinin inhibition assay. The antigen used for HI detection was LaSota strain.


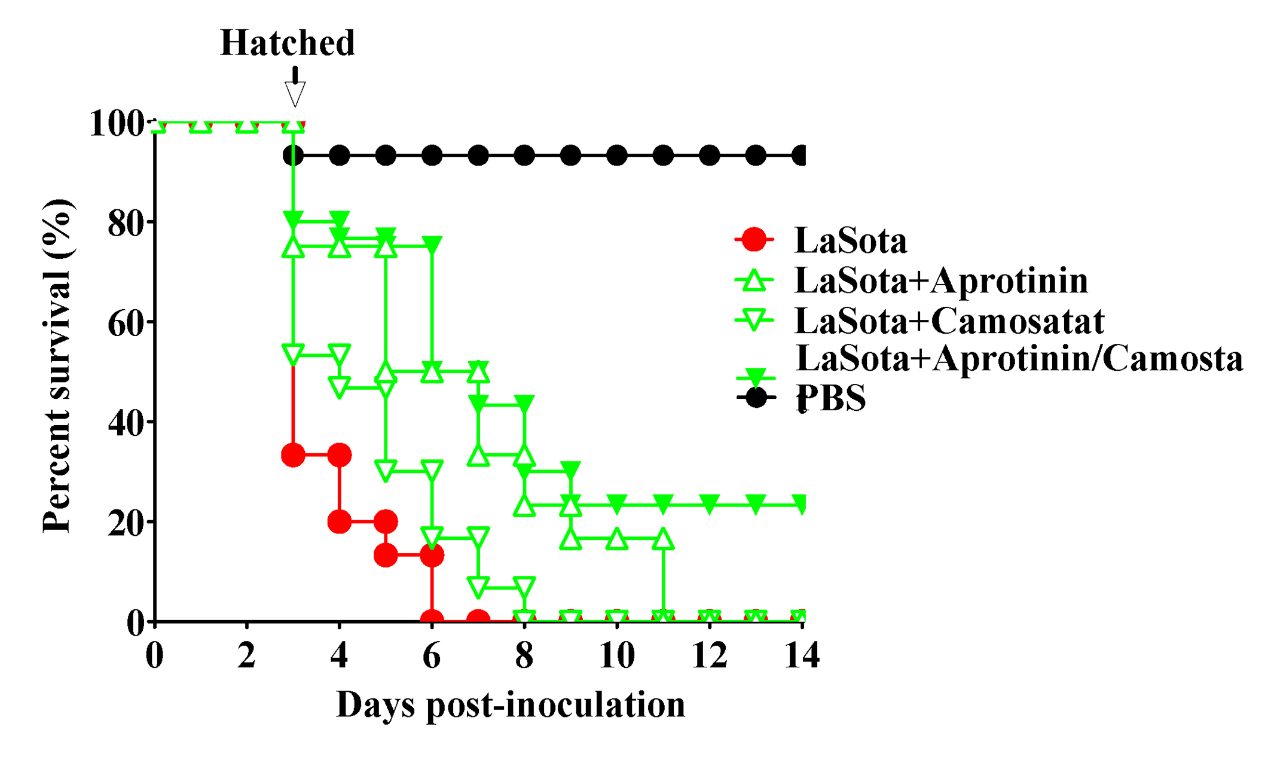


**FIG. S4. Effect of protease inhibitors on the pathogenicity of NDV strain LaSota in chicken embryos.** SPF chicken embryos were *in ovo* inoculated with NDV strain LaSota, with or without the addition of protease inhibitors in a 0.1 ml volume (10^3.0^ EID_50_ per egg) via the amniotic route at 18 embryo-days. The proportions of inoculated embryos that hatched successfully without assistance and survived to 14 days post-inoculation were recorded daily for each group.
